# Hashimoto's Encephalopathy and Seizure Disorders

**DOI:** 10.3389/fneur.2019.00440

**Published:** 2019-05-08

**Authors:** Jie Li, Fengzhen Li

**Affiliations:** ^1^Department of Neurology, The First Affiliated Hospital of Xinxiang Medical University, Weihui, China; ^2^Xinxiang Medical University, Xinxiang, China

**Keywords:** Hashimoto's encephalopathy, seizure, immunotherapy, autoimmune encephalopathy, diagnosis

## Abstract

Hashimoto's encephalopathy (HE) is a rare, clinically heterogeneous condition associated with positive thyroid autoantibodies. It is increasingly recognized as an important and treatable cause of autoimmune encephalopathy. Thyroid-associated antibodies such as thyroperoxidase (TPO) antibody, thyroglobulin (TG) antibody, and thyrotropin receptor (TR) antibody were found in HE patients with seizure disorders. Although antithyroid antibodies are required for the diagnosis of HE, their role in the pathogenesis of HE remains uncertain. Instead of playing a key role in the pathophysiology processes of HE, it is suggested that thyroid-associated antibodies are hallmarks of HE. Seizure disorders were found in approximately two-thirds of HE patients, and common anticonvulsant therapy alone is usually ineffective. Some patients did not respond to any antiepileptic drugs. The use of immunotherapy can effectively control seizure disorders. Electroencephalography and imaging findings are not specific to HE patients and can also be seen in other causes of encephalopathies. However, the prognosis in the majority of patients with HE was usually good if it is diagnosed and treated correctly.

## Introduction

Hashimoto's encephalopathy (HE) is a rare clinical condition first described by Lord Brain (1). The prevalence of HE in the adult population is estimated to be 2.1/100,000 subjects in a study examining patients with unexplained encephalopathy with detectable antithyroid antibodies (2). It has become increasingly recognized in the last few years as an important and treatable cause of autoimmune encephalopathy.

The incidence of HE is higher in female (about 70–88% of female patients). The average age of onset is about 40 years old (3). Clinically, there may be various manifestations such as seizure disorders, rapidly progressive cognitive impairment, and stroke-like attack. The course of the disease may be recurrence–remission or gradual progression. Characteristically, these patients usually have high titers of thyroperoxidase antibody (TPOAb) and respond well to corticosteroid therapy. Because of these features, an acronym, steroid-responsive encephalopathy associated with autoimmune thyroiditis (SREAT), was used in some research articles (4, 5). It was also called NAIM (non-vasculitic autoimmune inflammatory meningoencephalitis) because of the absence of cerebral vasculitis seen on brain biopsies in affected individuals (6). In fact, HE lacks a clear definition, and the symptoms often overlap with other neuronal antibody-associated autoimmune encephalopathies (7).

HE can be regarded as a possible immune encephalopathy due to its possible immune-mediated mechanism. The diagnosis criteria for HE remain a diagnosis of exclusion because its antibodies are not specific to HE patients. Thyroid antibodies and α-enolase antibodies (anti-NAE) have been detected in healthy people and patients with other autoimmune diseases. Although hundreds of HE patients have been reported in the literature, the specific mechanism of HE is not fully understood.

It is suggested that HE is better termed autoimmune encephalopathy associated with thyroid antibodies because antithyroid antibodies are essential laboratory features of the diagnosis of HE (8). Seizure disorders were seen in about 60–70% patients, and many of them showed as the first manifestation of the disease. HE was often misdiagnosed with other diseases by the neurologist and pediatrician, especially doctors not majored in epilepsy. Awareness of HE has increased in the last few years, but it is still rather uncommon. HE is easy to be misdiagnosed because of the low incidence and the atypical symptoms. However, if it is diagnosed and treated correctly, the prognosis of the disease is good. Therefore, it is important to recognize the characteristics of HE. In this article, we review the characteristics of seizure disorders and the diagnostics of HE.

## Clinical Presentation of Seizure Disorders in HE

HE is a rare, clinically heterogeneous condition with increased antithyroid autoantibodies (9). One report divided HE into two classes: vasculitic type and indolent progressive type; the former manifested with repetitive stroke-like events, such as transient hemiparesis, aphasia, and ataxia with no or only slightly cognitive impairment. The latter shows an insidious onset of altered consciousness, seizure attacks, hallucinations, or psychotic disorders. Seizures, tremors, myoclonus, and stupor can occur in both types (10). Unusual presentations of HE like headache and peripheral neuropathy were also reported in some cases (7, 11).

Seizures are common in patients with HE. Approximately two-thirds of patients of HE experience seizure disorders. Seizure presentations include progressive focal or generalized onset seizures and new-onset status epilepticus (SE) (12–14). SE includes epilepsia partialis continua (EPC), and non-convulsive SE (NCSE) has been reported in 12% of HE patients (9, 14, 15). The most common seizure pattern was focal onset seizures with secondary generalization (4, 16). Seizure disorders are more common in children (present with seizures about 80%) than in adults and change in level of consciousness (17, 18). The type of epileptic manifestation may be generalized or focal, convulsive as well as myoclonic. EPC, a form of SE characterized by recurrent seizures that can last for hours, days, or even longer can also be found in HE individuals (19). Varassi et al. (20) describe a man with recurrent episodes of unilateral left-sided auditory hallucinations. The patient did not respond to antiepileptic drugs, such as diazepam, levetiracetam, lacosamide, and phenytoin. The patient later developed a refractory NCSE presenting with a stuporous state. Visual hallucinations were also reported before the onset of seizures in HE patients (21). Presentation of faciobrachial dystonic seizures was reported in a 58-year-old patient diagnosed with HE. Screening of autoimmune antibodies especially voltage-gated potassium channels (VGKCs)/leucine-rich glioma inactivated 1 (LGI1) antibodies were negative. Instead, the finding of high titer of serum antithyroid and the dramatic response to steroid therapy led to the diagnosis of HE (9).

## Possible Mechanisms of Seizure Disorders in Hashimoto's Encephalopathy

The mechanisms of seizure disorders in HE are still not fully understood. Possible mechanisms including autoimmune mechanisms may play a variety of roles in the pathophysiology of epilepsy because HE belongs to a spectrum of autoimmune encephalitis (22). Thyroid-associated antibodies such as TPOAb, thyroglobulin antibody (TgAb), thyrotropin [thyroid-stimulating hormone (TSH)] receptor antibody (TRAb or TSHRAb), and α-enolase antibody targets for cortical neurons and endothelial cells were found in HE patients with epilepsy. Although antithyroid antibodies are important when HE is diagnosed, the role in the underlying pathogenesis mechanism remains unclear, and no direct correlation between serum antibody titers and clinical state of disease severity is found. The pathogenic roles of antibodies in HE have been questioned. Rather than playing a direct role in the pathophysiology of HE, it is suggested that thyroid-associated anti-TPO is a hallmark of HE (23). Yuceyar et al. reported a case with a family history, and they hypothesized that a genetic factor may participate in the pathogenesis of HE (24). Besides, other research suggested that toxic effects of TSH, brain hypoperfusion, and edema-induced cerebral dysfunction due to autoimmune-mediated vasculitis may also play a role in the mechanisms of seizure disorders (8, 25).

## Evaluation of Seizure Disorders in Patients With Suspected HE

For patients with suspected HE, diagnostic testing of blood and cerebrospinal fluid (CSF), electroencephalography, and neuroimaging such as brain computed tomography (CT) scan and magnetic resonance imaging (MRI) are important in differential diagnosis from other causes of neurologic disease, such as inflammation diseases, electrolyte and metabolic disturbances, multiple sclerosis, toxins, and tumors.

## Laboratory

Thyroid hormone dysfunction ranging from hypothyroid to thyrotoxic was found in HE. Most cases occur under euthyroid and hypothyroid metabolic conditions. TPOAb in serum is one of the most frequent signs of HE, ranging from several times to several 100 times higher than normal controls. Serum TgAb also increased (71%) in some patients; however, high-titer thyroid antibodies are not HE specific. They present in about 13% of healthy subjects and even higher (27%) in white women older than 60 years. Thyroid antibodies were also found increased in patients with other autoimmune encephalitis. Some scholars found NAE autoantibodies in the serum of patients with HE and considered that it may be a specific serological biomarker for the diagnosis of HE (25).

CSF examination may be needed in order to exclude other infectious or autoimmune encephalitis. Ilias et al. found that about 75% of individuals with HE presented with CSF antibodies, which are absent in the healthy individuals (26). The main changes of cerebrospinal fluid in patients were mild to moderate increase in protein and normal or elevated cerebrospinal fluid pressure. The increased rates in two studies were 78 and 66%, respectively (27). Lymphocytes can be slightly higher, sometimes with oligoclonal bands. Some patients might have other antibodies in addition to anti-TPO. Thus, it is necessary to detect all autoimmune antibody such as gamma-aminobutyric acid A receptor (GABAAR), N-methyl-D-aspartate receptor (NMDAR), LGi1, and antinuclear antibodies (ANA) (28).

## Neuroimaging

MRI findings of HE varied from normal to diversified appearance, including ischemic lesions, white matter demyelination, and focal vasogenic edema (29). Many studies showed that the CT/MRI imaging of HE may sometimes simulate an ischemic stroke, multiple tumors, granulomas, or even a degenerative disease (30–32). The diverse neuroimaging features of HE may be due to different or diverse pathological process stages of HE when performing CT/MRI scan. Various and mostly unspecific abnormalities were found by MR and/or CT in about 50%. Single photon emission computed tomography (SPECT) examinations showed attenuated cerebral perfusion in cortical areas or basal ganglia (33).

## Electroencephalography

The etiology of epileptic seizures includes structural metabolism, immunity, inflation, trauma, and endocrine and degeneration causes, among others. To clarify the causes of seizure disorders in patients with suspected HE, electroencephalography (EEG), laboratory examination of serum and CSF, MRI, SPECT, and neuropsychological examinations need to be used.

EEG is a useful tool in the evaluation of patients suspected of HE. Abnormal EEG results were recorded in 98% of patients with HE (27). Repeated EEG or long-term video EEG increased the positive rate of examination. EEG findings usually show moderate to severe abnormalities, which are often in parallel with clinical improvement after appropriate treatment (34). The EEG abnormalities seen in HE include non-specific diffuse slowing of the background activity (delta or theta frequency wave), interictal epileptiform discharges, repetitive focal spikes or sharps, photomyogenic response, photoparoxysmal response, and generalized biphasic or triphasic waves (35). Diffuse slowing of the background activity is the most common abnormality in HE individuals. The location of epileptic activity is not always consistent with the site of lesions shown on neuroimaging or physical examination (33, 36). Myoclonus seizures were found in about half of the patients with steroid-responsive encephalopathy associated with autoimmune thyroiditis in one study (36). None of these EEG findings were specific for the diagnosis of HE and can be seen in encephalopathy due to other causes. Because of the non-specificity of the EEG examination, it seems to be a limited tool in differential diagnosis of seizure disorders and/or encephalopathy with other possible causes of encephalopathy (37). However, EEG is helpful in reflecting changes in brain functions during hospitalization and follow-up.

## Diagnosis

Generally, the diagnostic criteria of HE are based on the clinical features with elevated antithyroid antibodies and good response to steroids (9).

When there are unexplained episodes of focal or generalized seizures, refractory to common antiepileptic drugs, with cognitive impairment and/or neuropsychiatric symptoms, Hashimoto encephalopathy may be considered. Before the diagnosis of HE is suspected on a patient with seizure disorders, detection of neural autoantibodies, lumbar puncture for CSF examination, and brain MRI/CT are needed to exclude other etiologies such as metabolic, infectious, vascular, and other inflammatory etiologies.

We should know that positive thyroid peroxidase antibodies and good response to steroid therapy are not sufficient criteria to establish the diagnosis of HE. Diagnostic criteria of HE have been proposed by Graus et al. (38) (Table 1) and Castillo et al. (4). These two criteria suggested that the diagnosis of HE remains a diagnosis of exclusion.

**Table 1:** Diagnostic criteria for Hashimoto's encephalopathy, from Graus et al. (38).

**Diagnosis can be made when all six of the following criteria have been met:**
1. Encephalopathy with seizures, myoclonus, hallucinations, or stroke-like episodes
2. Subclinical or mild overt thyroid disease (usually hypothyroidism)
3. Brain MRI normal or with non-specific abnormalities
4. Presence of serum thyroid (thyroid peroxidase, thyroglobulin) antibodies
5. Absence of well-characterized neuronal antibodies in serum and CSF
6. Reasonable exclusion of alternative causes

## Differential Diagnosis

Seizures are an extremely common symptom in HE and deserve consideration in the differential diagnosis of patients with newly onset epileptic seizures.

As all the diagnostic criteria have suggested, if the patients were diagnosed with HE, all the specific clinical syndromes of autoimmune encephalitis (with and without positive autoantibodies) and those accompanied by well-defined autoantibodies should be excluded. Due to the diversity of clinical manifestations of HE, some patients are prone to be misdiagnosed as having viral encephalitis because of their prominent psychiatric symptoms. Prominent symptoms of cognitive dysfunction, tremor, and seizure are easily misdiagnosed as Creutzfeldt-Jakob disease (CJD). If HE is characterized by a stroke-like episode, it needs to be differentiated from central nervous system vasculitis. Therefore, when clinically highly suspected to be CJD, the possibility of HE should be considered. In the literature, 53% of patients initially diagnosed with CJD were eventually diagnosed with HE (8); at the same time, HE should be distinguished from primary mental disease, metabolism, poisoning, and paraneoplastic encephalopathy. If the patients combined with peripheral nerve damage, Guillain–Barre syndrome should be excluded. Patients with HE may also have a positive ANA, thus often causing confusion with neuropsychiatric involvement in systemic lupus erythematosus. We should pay attention to the fact that psychiatric symptoms can also occur in patients diagnosed with hypothyroidism.

## Treatment

Once the diagnosis of HE is made, immunotherapy usually brings a dramatic recovery. Seizure disorders accompanied with HE are usually refractory to antiepileptic drugs unless immunotherapy was used. Common anticonvulsant therapy alone is usually ineffective; some patients did not respond to any antiepileptic drugs, including valproic acid, phenytoin, levetiracetam, lacosamide, topiramate, midazolam, and even propofol (20). The use of immunotherapy in the acute stage of HE not only can effectively control seizure disorders but also can assist in the diagnosis of immune epilepsy.

High-dose glucocorticoids and intravenous immunoglobulin are the first-line treatment of HE. First-line treatment also includes plasma exchange. When the first-line treatment regimen is ineffective or has a poor response, second-line treatment (including rituximab and cyclophosphamide) can be used. Patients who received early immunotherapy usually had a better prognosis. Study showed that patients receiving second-line treatment also had a better prognosis than those who did not receive second-line treatment when the first-line treatment was ineffective (7). When the disease is in a stable state, the immunosuppressive agent will be kept in the lowest effective dose for a while and then tapered slowly (39). Steroid treatment leads to complete neurological recovery in most patients, but patients will not always be responsive to corticosteroids. For these patients, other alternative forms of immunity therapy should be tried.

Seizures and other neurological features can also improve dramatically after intravenous immunoglobulin and plasmapheresis, alone or in combination (9, 33). Cyclophosphamide or rituximab can be used as a second-line medication when it is encountered in patients with refractory epilepsy. In recent years, it has been found that T cell inhibitors (cyclosporine A, tacrolimus, and sirolimus) successfully applied to control seizures. Others such as methotrexate, azathioprine, and hydroxychloroquine also showed effectiveness in reported cases (40).

Often, antiepileptic drug therapies that control seizures do not need to be used in the long term in patients with HE. It should be mentioned that seizure disorders can recur especially when steroids were tapered; hence, in some patients, maintenance immunotherapy is necessary (21). For patients with recurrent symptoms, reuse of glucocorticoids, plasma exchange, or immunoglobulin therapy is still effective (33). In order to prevent recurrence, it is recommended that glucocorticoid therapy should be done in sufficient maintenance doses and tapered slowly. Second-line immunosuppressant drugs mentioned above can be used if necessary. It is inappropriate to use serum TPOAb as a marker to determine when steroid therapy should be stopped because the effect of corticosteroids on TPOAb serum levels remains controversial (2, 41). For those patients with severe sequelae, including cognitive impairment and refractory seizures, immunotherapy, and antiepileptic drugs should be used longer (42, 43). Use of immunotherapy requires a close follow-up and regular measures for prevention of side effects.

## Conclusion

Seizure disorders are common manifestations of HE. The diagnosis of HE still mainly depends on clinical presentation and supplementary examinations (including EEG, CT and/or MRI, and neuroelectrophysiology). The exact molecular mechanism that leads to seizures is still not clear. This type of immune-related seizures is not sensitive to conventional antiepileptic drugs, but has obvious effects on immunomodulatory therapy. Immunosuppressive therapy should be used in addition to antiepileptic drugs to control seizure disorders when HE is diagnosed. However, a better prognosis can be achieved when diagnosed early and treated with immunotherapy. We suggest that the diagnosis of HE should be considered in patients with unexplained encephalopathy presenting with uncontrolled seizures because steroid therapy is highly efficacious in these patients and is reversible.

The clinical spectrum of autoimmune epilepsy syndromes is expanding. HE is a rare, progressive, and relapsing multiform disease. Numerous challenges remain with the diagnosis and exploring the mechanisms of HE. A better understanding of the specific mechanisms underlying autoimmune epilepsy in HE is needed in the future.

## Author Contributions

All authors listed have made a substantial, direct and intellectual contribution to the work, and approved it for publication.

### Conflict of Interest Statement

The authors declare that the research was conducted in the absence of any commercial or financial relationships that could be construed as a potential conflict of interest.
